# Determination of the Long-Term Thermal Performance of Foam Insulation Materials through Heat and Slicing Acceleration

**DOI:** 10.3390/polym14224926

**Published:** 2022-11-15

**Authors:** Minjung Bae, Hosang Ahn, Jaesik Kang, Gyeongseok Choi, Hyunjung Choi

**Affiliations:** 1 Department of Building Energy Research, Korea Institute of Civil Engineering and Building Technology, Goyang 10223, Republic of Korea; 2 Department of Smart Convergence Architecture, College of Engineering, Ajou University, Suwon 16499, Republic of Korea

**Keywords:** acceleration methods, aging, thermal resistance, extruded polystyrene, polyisocyanurate, phenol foam

## Abstract

Foam insulation materials are widely used in the construction industry due to their low thermal conductivity attributable to their microstructures and their low-conductivity blowing agents and affordability. In this study, we evaluate how the thermal performance of foam insulation materials used for the exterior walls of buildings, viz., extruded polystyrene (XPS), polyisocyanurate (PIR), and phenolic foam (PF), age over the life cycle of a building. To compare the aging of thermal performance during the life cycle of a building, each material was tested at 70 and 110 °C and with slicing acceleration according to EN and ISO standards. The thermal conductivity of each foam insulation material was measured using a heat flow meter at an operating temperature of 23 °C and converted into thermal resistance values. Different foam insulation materials have different aging procedures according to material-specific EN standards, while ISO 11561 applies the same procedure to all material classifications. Upon comparing the aged values according to ISO and EN standards to the initial values, the analysis showed a change rate of 23 to 26% in PIR and 18 to 20% in PF. In XPS, a rate of change of 10 to 23.8% was calculated. Our results indicated that the slicing acceleration induced a thermal resistance reduction rate about three times faster than aging at 70 °C. However, the long-term changed thermal resistance values of the foam insulation material applied via the calculating procedure specified in the ISO and EN standards were similar.

## 1. Introduction

With the acceleration of urbanization, the increased energy requirements for lighting, refrigeration, ventilation, and the heating and cooling systems of buildings has increased the consumption of natural resources. Many countries are implementing systematic plans to satisfy goals for a reduction in greenhouse gas emissions in the building sector in accordance with strategies promoting carbon neutrality. Using building insulation materials is one of the most effective measures to reduce heating and cooling losses through the exterior wall [1]. Organic insulation materials are used in many buildings due to their low thermal conductivity and relatively low cost [2], and foam insulation materials, such as polystyrene (PS), polyurethane (PU), and phenol foam (PF), make up most of the building insulation market. In order to support a reduction in greenhouse gases in the building sector, it is necessary to consider the evaluation method and quality standards considering the aging characteristics of building insulation.

It is necessary to study how much the thermal performance of foam insulation materials such as extruded polystyrene (XPS), polyisocyanurate (PIR), and phenol foam (PF) used in building envelopes can deteriorate over the service-life of a building. The effect of aging on the thermal performance of insulating materials has been analyzed by elucidating the influence of temperature, humidity, and apparent density on their thermal conductivity [3,4,5]. Insulation materials generally consist of a solid matrix material with gases randomly or regularly dispersed within cells, pores, and cavities [6,7]. In foam insulation materials, thermal conductivity is determined by the fineness and distribution of cells and, in particular, the gases they contain. However, the mechanical and thermal properties of insulating materials change significantly over time. The blowing agent remains inside the cell after a foam insulation material is manufactured and is replaced by air over time [8,9]. One of the aging effects which has the greatest impact on thermal conductivity is the replacement of the highly insulating blowing agents with oxygen from the environment, accelerating the absorption of moisture [10,11,12].

In the initial stage of aging, the diffusion of air inside the cell structure occurs faster than that of the blowing gas, leading to a rapid decrease in thermal conductivity [13]. Because the thermal conductivity of the remaining blowing agent is lower than that of air, a very slow decrease in thermal conductivity is observed as equilibrium is established between the diffusion of air and the blowing agent inside the cell structure blowing gas. At temperatures close to room temperature, the aging process can last for decades, the rate of which depends on the type of polymer used and the gas and foam filling conditions.

Therefore, methods that accelerate the aging process are desirable for various reason. First, the rated thermal property is used for comparison for building insulation. It is useful if the rated value reflects the long-term performance of each insulation material. Additionally, it enables the architect to predict the thermal performance of the building over its service-life, and be used to plan HVAC systems for heating and cooling in the interior.

The aging process adopted in the relevant literature can consist of a heat acceleration method to increase the diffusion rate of the foaming agent and a slicing acceleration method to reduce the diffusion length of the foaming agent. European and international standards already operate by adopting heat and slicing acceleration methods on the aging insulation material.

Changes in long-term thermal properties caused by the aging of insulating materials have been noted in the relevant literature. To mention a recent study, Berardi et al. [14] investigated the temperature- and humidity-dependence of the thermal conductivity of PU and PIR, revealing that high temperature accelerates the diffusion process and high humidity destroys solid materials. Makaveckas et al. [15] analyzed the thermal conductivity of PIR products aged under four temperature conditions and reported varying thermal conductivities according to thickness and degree of diffusion prevention by surface skin. Winkler-Skalna et al. [16] performed an aging procedure of the PUR foam in accordance with the EN standard and reported the effect of the apparatus density and mean temperature in a test on the change in thermal conductivity. Choi et al. [17] analyzed the thermal properties of PS using a standard test method that intentionally increases the release rate of the blowing agent using slicing materials.

As described above, the previous studies were conducted on the aging phenomenon of foam materials. However, comparative studies about foamed materials, including studies on thermal properties that deteriorate during the service-life of a building, are still rare. In addition, the diversity of aging procedure conditions previously adopted makes it difficult to compare research results.

In this study, a standardized aging procedure in accordance with EN and ISO was followed to compare aging thermal performance on foam insulation materials for buildings. The EN standard describes thermal insulation products for buildings; EN 13164 [18] specifies XPS, EN 13165 [19] specifies rigid polyurethane foam (PU), and EN 13166 [20] specifies PF product specifications. These standards define the heat and slicing acceleration method according to product characteristics. Meanwhile, ISO 11561 [21] specifies an accelerated slicing test method for closed-cell cellular plastic materials.

Another purpose is to examine the variability of thermal performance according to various aging procedures. All accelerated test methods can predict the thermal degradation of foam insulation materials over the life of a building (generally assumed to be 25 years), and their expected aging values may differ moderately. The proper selection of an aging procedure is pivotal in the quantitative estimation of the change in thermal conductivity of a foam insulation material due to aging. This study analyzed the aged value of each aging procedure applied to the same foam material.

Thermal conductivity depends on three main factors: operating temperature, moisture content, and density [22,23,24]. From a series of empirical observations for some insulating materials, including expanded polystyrene, XPS, and PU, [25] the authors showed that the relationship between the effective thermal conductivity and temperature is linear. However, for certain foam materials, this relationship is non-linear and difficult to predict because the thermal conductivity increases at low temperatures [9,26,27].

The thermal conductivity (thermal resistance) of foam insulation materials is generally tested in accordance with KS F 9016 [28], which cites both ISO 8301 [29] and ISO 8302 [30]. The European Standard EN 12667 [31] also cites the same international standards. However, the thermal conductivity of these materials can respond differently to operating temperatures, and there is no uniform test condition that allows direct comparison between insulating materials [12,32]. The thermal conductivity of a foam insulation material is usually declared by the manufacturer for standard laboratory conditions, for example, a standardized average temperature around 23 °C and 50 ± 10% relative humidity (R.H.). The current study was carried out to streamline the comparison by applying the same standardized average temperature and R.H. used in measuring the aged thermal conductivity of insulating materials.

## 2. Materials and Methods

### 2.1. Description of the Aging Procedures

The accelerating procedures were applied according to the appropriate European Standard for each foam insulation material. EN 13165 (XPS) prescribes that if the thickness of the sample is between 20 and 70 mm, it should be sliced into 10 ± 1 mm specimens and stored at 23 ± 2 °C and 50 ± 5% R.H. for 90 ± 2 days. The aged slices are then assembled and the aged thermal conductivity measured.

According to EN 13165 (PIR), the aged thermal conductivity is determined using two methods. The first method is the direct measurement method (accelerated-aging procedure). The sample should be stored at 70 ± 2 °C for 175 ± 5 days, and a safety factor added to the measured value. The second method is a combination of the normality test and calculation method (fixed-increment procedure). The sample is kept at 70 ± 2 °C for 21 ± 2 days and the thermal conductivity measured. If the difference between the initial and measured values exceeds the criteria for a specific blowing agent, a fixed increment is added to the initial thermal conductivity value to calculate the aged thermal conductivity.

The EN 13166 (PF) standard describes two methods for the determination of the aged values: Method 1 (slicing) and Method 2 (heat aging). In Method 1, the test sample is cut into 10 ± 1 mm thick slices, and the initial value of the thermal conductivity measured. These specimens (slices) are kept at 23 ± 2 °C and 50 ± 5% R.H. for a period depending on the thickness of original specimen. Cutting the test sample into slices increases the open cell content exposed on the surface. To correct for the reduction in effective thickness, the initial thermal conductivity of the sample is subtracted from the initial thermal conductivity of the assembled slices. The accelerated value of thermal conductivity according to Method 1 is reported as the thermal conductivity value obtained for the assembled slices minus the correction for the damaged surface layer. In Method 2, the sample should be aged at 70 ± 2 °C for 175 ± 5 days. Alternatively, the test sample should be conditioned at 70 ± 2 °C for 7 days, and then, aged at 110 ± 2 °C for 14 ± 1 days.

Thus, the European Standard aging procedures for XPS, PIR, and PF are different. This study sought to consider a standardized aging procedure that can be generally applied to all foam insulation materials. ISO 11561 prescribes the determination of the long-term decrease in thermal resistance (aging) of closed-cell cellular plastic materials and products based on a slicing procedure. This standard concerns the possibility that the material itself may change under high-temperature conditions, not gas diffusion. It describes two aging procedures: slicing and Method B, which is a simple test method for determining the service-life (25 years or more) of the thermal performance of a closed-cell foamed plastic product without surface treatment.

**Figure 1:**
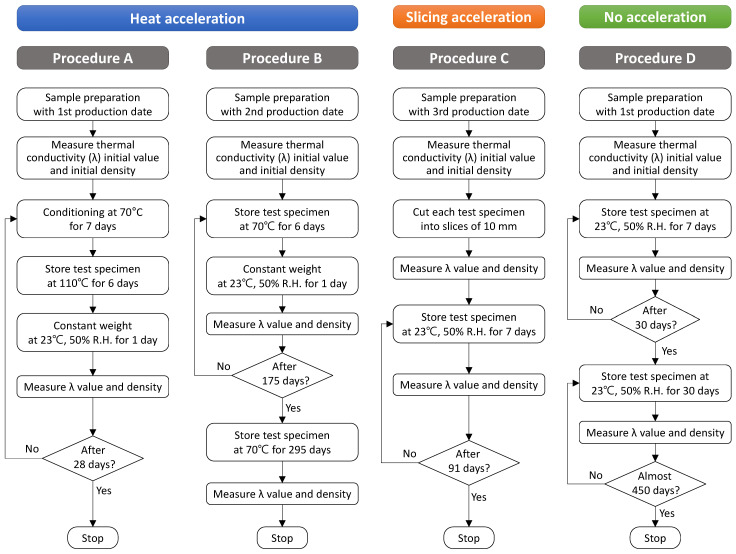
Heat and slicing acceleration procedures following EN and ISO standards.

In order to efficiently perform the acceleration method specified in the EN and ISO standards mentioned above, four types of aging procedures were set up for measuring the aged thermal conductivity of a foam insulation material, as shown in Figure 1.

This study analyzes the differences in the long-term thermal performance of building insulation materials according to the acceleration adopted. For this purpose, heat acceleration at either 70 or 110 °C and slicing acceleration procedures were applied to all the samples, but the aging value of each foamed insulation material was calculated according to the applicable standard of each. Procedure A reflected the heat acceleration at 110 °C in EN 13166 (PF), and the aging period was 28 days.

Procedure B reflected the heat acceleration at 70 °C in EN 13165 (PIR) and EN 13166 (PF). The test period was 295 days, and the thermal conductivity and density of the samples were tested weekly until 175 days. After the samples were stored at 70 °C for another 120 days, the thermal conductivity and density of each were tested again. Procedure C involved slicing acceleration and reflected EN 13164 (XPS), EN 13166 (PF), and ISO 11561. EN 13164 (XPS) and ISO 11561 prescribe a test period of 90 ± 2 days, while EN 13166 (PF) specifies a test period depending on the thickness of a sample. The test period of Procedure C was set to 90 ± 2 days, and the thermal conductivity and density of each assembled sample were tested weekly.

In Procedure D, which was set up as the control, the samples were just stored at 23 ± 2 °C and 50 ± 5% R.H. for 450 days. Samples were cut from products with three different production dates, and the start date of each accelerated test was set as close as possible to the production date. The thermal conductivity and density were tested weekly until 30 days and tested once a month thereafter until the end. The thermal conductivity of all samples was tested according to ISO 8301 and ISO 8302 using heat-flow meter apparatus (HFM 436/3/1E, NETZSCH, Selb, Germany).

### 2.2. Description of the Test Samples

This study was conducted on XPS, PIR, and PF, and all samples were obtained from two manufacturers selected for each material. In Korea, foam insulation materials should satisfy appropriate standards such as KS M 3808 (PS) [33], KS M 3809 (PU) [34], and KS M ISO 4898 (rigid cellular plastics) [35]. As shown in Table 1, two types each of XPS and PIR and one type of PF that obtained quality certification by KS standards were selected. XPS uses HCFC (141b, 142b) as a forming gas, PIR uses cyclopentane, and PF uses a mixture of isopentane and cyclopentane for foaming.

Owing to a delay in the supply of one of the PF samples, only the last aging procedure was conducted for both PF samples. Procedure D was applied to both PF samples but the storage periods differed. Procedure D was applied to a random selection of manufacturers who provided XPS and PIR samples for Procedures A to C. All samples were cut to a size of 300 × 300 mm^2^, and the XPS and PIR samples had thicknesses of 50 and 90 mm, respectively, and the PF samples had thicknesses of 60 and 70 mm. Table 2 shows the definitions of the sample names.

## 3. Results

### 3.1. Thermal Resistance

For all samples of foam insulation materials, the thermal conductivity was measured every 7 days using a heat flow meter to compare and analyze the effect of the heat acceleration (A and B) and slicing acceleration (C) aging procedures.

**Figure 2:**
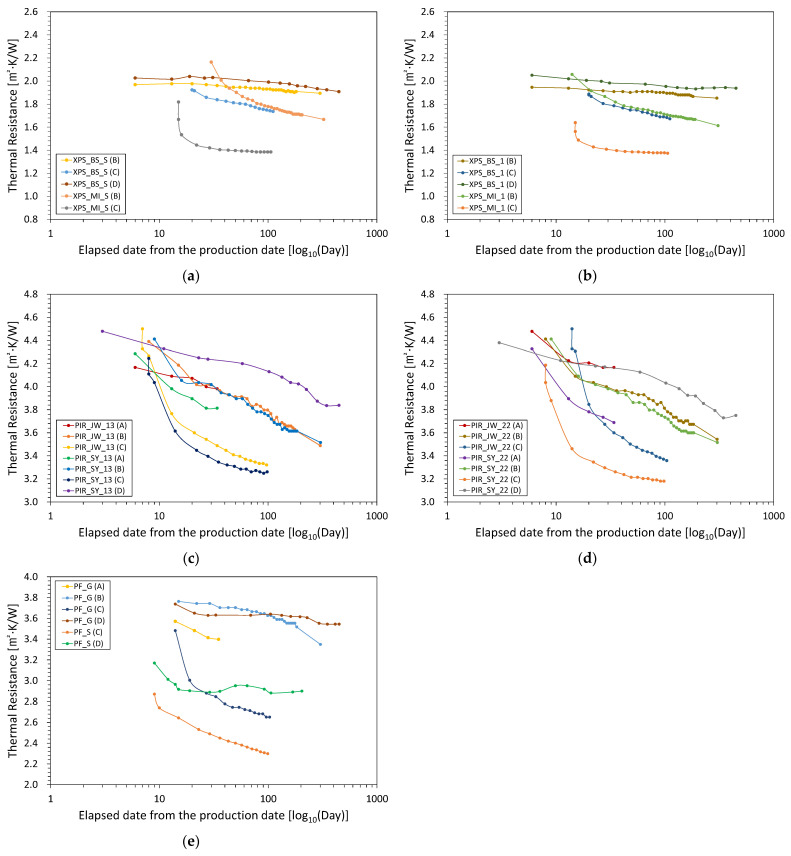
Changes in the thermal resistance of (**a**) XPS No. S, (**b**) XPS No. 1, (**c**) PIR 1st class—No. 3, (**d**) PIR 2nd class—No. 2, and (**e**) PF 1st class—No.1 according to each aging procedure.

The purpose of this study is to compare the aged thermal properties of various foam materials by applying the same acceleration method. Thermal conductivity is advantageous for explaining heat transfer characteristics through completely different materials with the same thickness. However, it is difficult to easily compare the thermal properties of foam materials with various thickness. In this study, the reciprocal values obtained by dividing the thickness by the thermal conductivity were calculated as the thermal resistance, and these are denoted in Figure 2. Since the effect of the thickness variable on the thermal performance is excluded, a more direct comparison is facilitated.

The thermal resistance measurement date represents the elapsed time after the production date, making it possible to compare the change in thermal resistance due to aging on the same time axis for foam insulation materials with different production dates. First, the measured thermal resistance values 28, 91, and 175 days after the start of Procedures A to C were compared. Then, the acceleration rates via heating or slicing were reviewed in comparison to the results from Procedure D.

**Figure 3:**
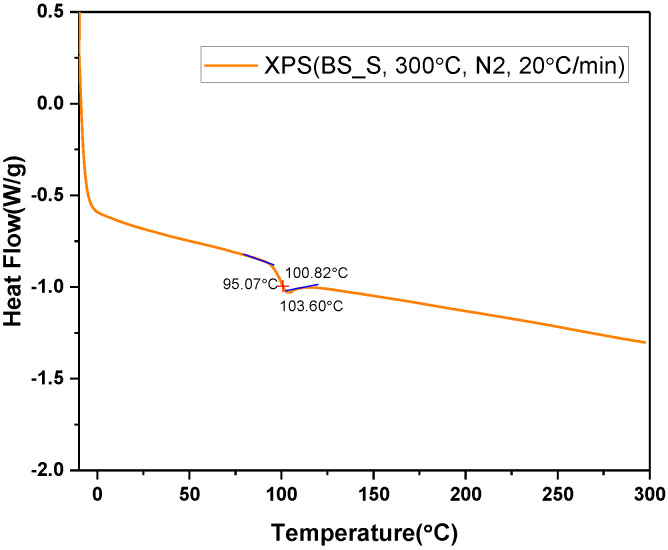
The differential scanning calorimetry (DSC) curve on XPS_BS_S sample.

The change in thermal resistance of the XPS samples is shown in Figure 2a,b. Because the samples were deformed at 110 °C, the thermal resistance could not be obtained according to Procedure A. In order to analyze the state change of the XPS sample according to temperature, differential scanning calorimetry (DSC) was used on XPS_BS_S, as shown in Figure 3. At the beginning of the test, the DSC curve showed a characteristic shift in the endothermic direction. The transition starts to show at 95 °C, and after the transition, the curve is almost horizontal. It appears that Procedure A causes the glass transition in the XPS sample, making it different from the original material.

The application of Procedure B to XPS_BS_S and XPS_BS_1 induced very small changes in their thermal resistance after 28 days, and decreases of 2.0–2.3% and 3.5–3.9% after 91 and 175 days, respectively. The start date of Procedure B for XPS_MI_S was 30 days after the production date, and the thermal resistance of XPS_MI_1 decreased by 10.3% after 28 days. In addition, the thermal resistance of both XPS_MI samples decreased by 19.3–21.2% and 23.0–26.4% after 91 and 175 days, respectively, with a larger reduction rate observed for XPS_MI_S than XPS_MI_1. Applying Procedure C, the thermal resistance decreased significantly when the assembly was tested immediately after slicing. The thermal resistance of XPS_BS_S decreased by 3.3% and 8.8% after 28 and 91 days, respectively, while that of XPS_BS_1 decreased more rapidly to 4.3% and 10.5%. In addition, Procedure C induced larger decreases in the thermal resistance of the XPS_MI samples than those of the XPS_BS samples. The thermal resistance of XPS_MI_S decreased by 21.9% and 23.8% after 28 and 91 days, respectively, and that of XPS_MI_1 decreased by 14.1% and 16.0%

The results of aging the PIR samples are shown in Figure 2c,d. After 28 days of applying Procedure A, the thermal resistance of PIR_SY_13, PIR_JW_13, PIR_SY_22, and PIR_JW_22 decreased by 11.0%, 4.0%, 13.7%, and 6.9%, respectively. In comparison, after 28 days of applying Procedure B, the thermal resistance of the PIR first class—No.3 samples decreased by 9.3–9.8%, while that of the second class—No.2 samples decreased by 10.3–10.8%. After 91 days, the thermal resistance of the PIR_SY samples decreased by 17.2–17.6%, while that of PIR_JW_13 and PIR_JW_22 decreased by 15.6% and 14.2%, respectively. Even after 175 days, the PIR_SY samples showed a similar rate of degradation (22.1–22.5%) regardless of the product type, and PIR_JW_13 (21.0%) showed a slightly lower thermal resistance than that of PIR_JW_22 (20.1%). By applying Procedure C, the decrease in the thermal resistance occurred very quickly before 28 days, and the decrease rate (20.0–21.3%) after 28 days was the highest of all the acceleration methods. After 91 days, the thermal resistivity of the PIR_SY samples decreased by 23.5–24.0%, while that of the PIR_MI samples decreased by 25.1–25.9%.

For the PF_G sample, the thermal resistance decreased by 11.1%, 0.5%, and 17.3% after 28 days in Procedures A, B, and C, respectively (Figure 2e). The reduction in thermal resistance after 28 days of Procedure C was higher than the decrease (5.6%) after 175 days of Procedure B. In addition, the rate of decrease in thermal resistance after 91 days in Procedures B and C was 3.1% and 17.3%, respectively. For PF_S, only Procedure C was applicable, and the thermal resistance decreased by 13.3% and 19.6% after 28 and 91 days, respectively. Therefore, the reduction in thermal resistance due to the application of Procedure C was slightly greater than that of the PF_G sample.

Procedure D was performed on certain samples and changes in the thermal resistance were compared to those of the acceleration rates due to heating and slicing. The procedure was carried out at the same time that Procedure A was started and was carried out for 450 days. After the test, the thermal resistance of XPS_BS_S and XPS_BS_1 decreased by 5.8% and 5.5%, respectively. For XPS_BS, even when aging was accelerated at 70 °C, the change in thermal resistance was not faster than when the sample was stored at room temperature. Similarly, after 450 days, the thermal resistance of PF_G decreased by 5.2%. Therefore, even if aging was accelerated at 70 °C, the change in thermal resistance was not faster than if the sample was kept at room temperature. Because the PF_S sample arrived late, Procedure D was only carried out for approximately 200 days. After 175 days, the thermal resistance of S_PF decreased by 8.5% and remained unchanged until the end of the test.

In summary, the 110 °C thermal acceleration (Procedure A) for the foam insulation materials (except for the XPS samples) was too short a period for the change in thermal resistance to stabilize. Thermal acceleration at 70 °C (Procedure B) decreased the thermal resistance of the PIR samples by 20.1–22.5% after 175 days. However, for some XPS and PF samples, the induced decreases in thermal resistance were smaller than 10%, which is similar to the aging value after 450 days of storage at room temperature (Procedure D). Therefore, heat acceleration did not lead to sufficient stabilization of the aging values for all materials. The slicing method (Procedure C) decreased the thermal resistance of the PIR samples by 23.5–25.9% after 91 days and led to minimal decreases in the thermal resistance of the XPS and PF samples, even with 70 °C thermal acceleration. For the XPS sample, the thermal aging value stabilized, while that of the PIR sample was also relatively stable. PF may still change if the test is conducted for more than 91 days.

### 3.2. Density and Relative Weights

**Figure 4:**
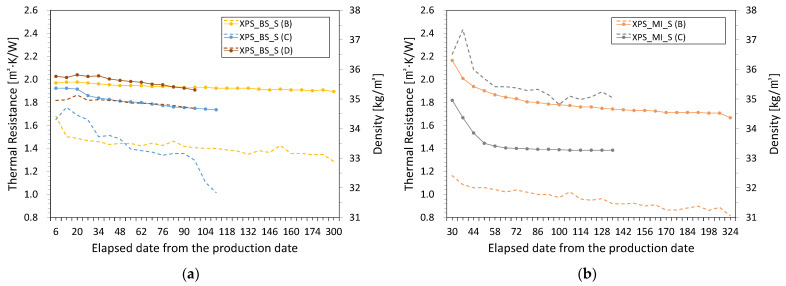

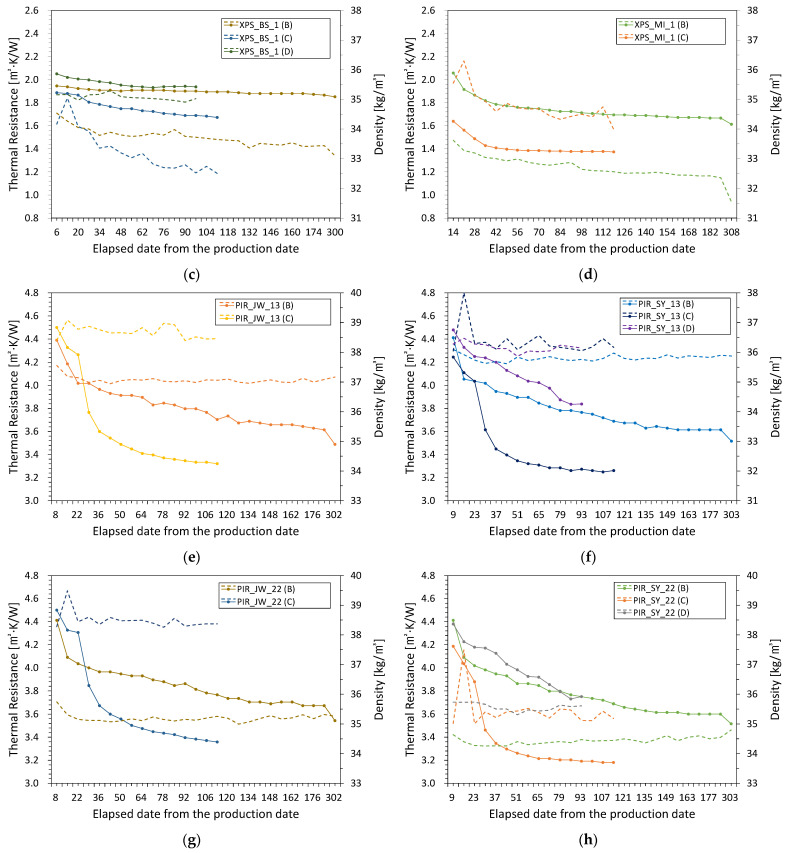

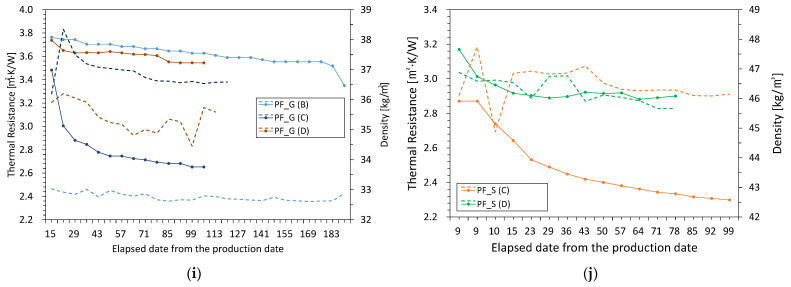
Changes in the density and thermal resistance of (**a**) XPS_BS_S, (**b**) XPS_MI_S, (**c**) XPS_BS_1, (**d**) XPS_MI_1, (**e**) PIR_JW_13, (**f**) PIR_SY_13, (**g**) PIR_JW_22, (**h**) PIR_SY_22, (**i**) PF_G, and (**j**) PF_S according to each aging procedure. The dots and solid lines in each graph represent thermal resistance according to the sample and aging procedure, and the dotted lines mean apparent density.

**Figure 5:**
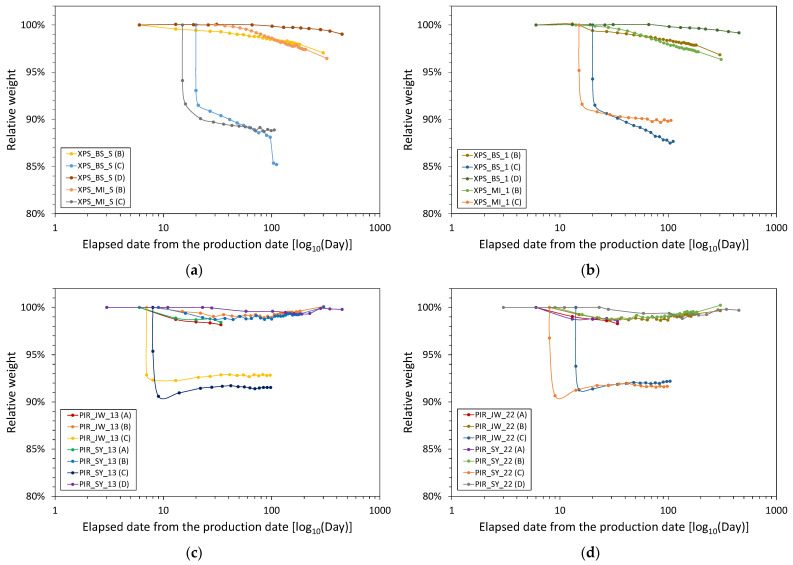

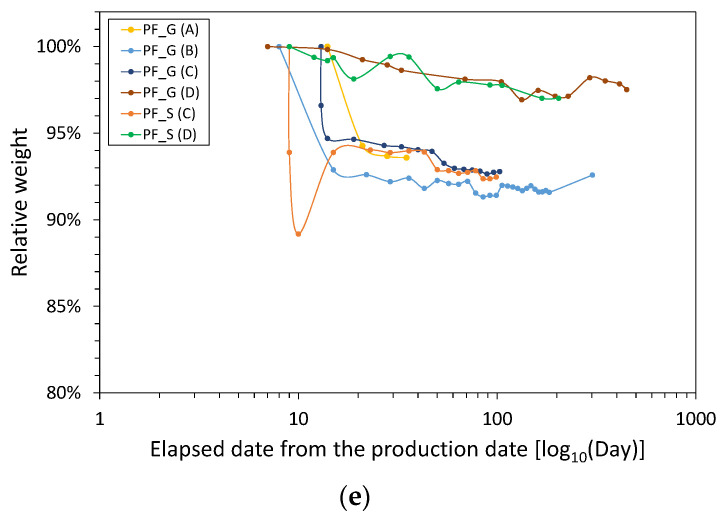
Changes in the relative weight of (**a**) XPS No. S, (**b**) XPS No. 1, (**c**) PIR 1st class—No. 3, (**d**) PIR 2nd class—No. 2, and (**e**) PF 1st class—No. 1 according to each aging procedure.

Figure 4 shows the variation in the density and thermal resistance of the foamed insulation materials aged via the application of Procedures B to D. Although Procedure A could be applied to all foam insulation materials except for the XPS samples (deformation occurred), the results are excluded in Figure 4 to focus on long-term changes. The density of all samples decreased and most samples lost their initial weight during aging. Figure 5 shows the weights of the samples during aging relative to their initial weights. The abscissa of the graph is again plotted on a logarithmic scale, as in Figure 2. The initial average densities of all XPS and PIR samples were 34.5 and 36.3 kg∙m^−3^, respectively. For PF, the initial average densities of the samples from the L and S manufacturers were 35.6 and 46.5 kg∙m^−3^, respectively. The rate of change in density 28, 91, and 175 days after the start date of each acceleration procedure was compared to compare the effects of heat and slicing acceleration.

The density of the XPS samples decreased during aging, as shown in Figure 4a–d. In Procedure B, the density of XPS_BS_S remained almost the same after 28 days and decreased by 1.9% and 3.4% after 91 and 175 days, respectively. Similarly, the density of XPS_BS_1 decreased by 1.5%, 2.3%, and 3.7% after 28, 91, and 175 days, respectively. The production date of XPS_MI_S was 30 days after the start date of Procedure B, while the density of XPS_MI_1 decreased by 1.3% after 28 days. After 175 days, the density of all the XPS_MI samples had decreased by 3.6%. The change in density was relatively consistent with the change in relative weight for the XPS samples. The relative weight of the XPS samples decreased to 97.2–98.0% after 175 days, and at the end of the test, the relative weights of XPS_BS_S, XPS_BS_1, XPS_MI_S, and XPS_MI_1 were 97.1%, 96.8%, 96.5%, and 96.4%, respectively. As a result of the slicing acceleration method (Procedure C), the XPS_BS samples maintained the same initial densities for 28 days, which decreased by 3.3% and 4.0% for PS_BS_S and XPS_BS_1 after 91 days, respectively. Furthermore, the slope of the change in density of XPS_MI_1 over time was gradual and increased steeply in the order of XPS_MI_S, XPS_BS_1, and XPS_BS_S. This order is opposite to that obtained for Procedure B. After 91 days, the relative weights of XPS_BS_S, XPS_BS_1, XPS_MI_S, and XPS_MI_1 were 85.2%, 87.7%, 88.9%, and 89.9%, respectively. These relative weight values were higher than those at the end of Procedure B. Therefore, the slicing acceleration method promotes weight loss compared to heat acceleration.

As a result of Procedure A, the densities of the PIR first class—No.3 samples changed by 2.3–2.4% after 28 days, while those of PIR_SY_22 and PIR_JW_22 changed by 1.2% and 2.8%, respectively. In Procedure B, the densities of the PIR_SY samples were 1.1–1.3% lower than the initial values after 28 days, and those of the PIR_JW samples were 1.5–1.7% lower. The densities of PIR_SY_13 and PIR_SY_22 were barely changed after 91 days and 175 days. After 28 days, the densities of PIR_JW_13 and PIR_JW_22 changed by 1.5% and 1.7%, respectively, and changed negligibly until 175 days. At the end of the test, the density of PIR_JW_13 was 1.0% lower than its initial value and that of PIR_JW_22 was slightly higher. The densities of all PIR samples in the heat acceleration method had changed by about 1% to 2%, and like in the case of the XPS samples, there was no noticeable increasing or decreasing pattern. As shown Figure 5c,d, all PIR samples had little change in relative weight with the heat acceleration method. The dimensions of the PIR samples did not change and the changes in their relative weights was reflected in the changes in their densities.

After 28 days of Procedure C, the densities of all the PIR samples were greater than those of the samples before slicing, and the densities of the PIR_SY samples changed more than those of the PIR_JW samples. After 91 days, the densities of the PIR_SY samples were similar to those after 28 days, while those of the PIR_JW samples were comparable to their initial values. The dimensions of the PIR samples did not change during Procedure C. All the PIR samples had a lower weight immediately after slicing than at the end of the test.

The density of PF_G in Procedure B decreased by 0.5%, 1.1%, and 1.2% after 28, 91, and 175 days, respectively (Figure 4i,j). In procedure C, the density of the PF_G sample increased by 2.5% before slicing after 28 days, and after 91 days, there was only a very small difference from the initial density. This is because the weight started to increase again after a rapid weight loss, as was observed for Procedure B.

### 3.3. Aged Thermal Resistance Value According to Standards

In general, the aging value of foam insulation materials can be calculated according to Method B in ISO 11561, and specifically according to EN 13164, EN 13165, and EN 13166 for XPS, PIR, and PF samples, respectively. The thermal resistance of each foam insulation material before and after the application of a specific aging procedure was calculated, and the aging values compared. In addition, we attempted to predict the rate of change in thermal resistance according to a specific standard method when heating and slicing were applied for a certain period. Table 3 summarizes the initial and aging thermal resistance values according to ISO, EN, and Procedures A~D for each sample. Procedures A~D were intended to compare the initial value against the measured thermal resistance at the end of the heat and slicing acceleration, and the effect of the calculation procedure specified in the ISO and EN standards on the declared value could be compared.

In the PIR samples, the aging values calculated according to the ISO and EN standards were found to have a rate of change of 23~26% compared to the initial values. The calculated aging values of the PF samples were found to have a rate of change of 18~20% compared to the initial values. However, for the XPS samples, a rate of change of 10~23.8% was determined. This appeared to be different depending on the manufacturer, and there was a difference in the rate of change even in products from the same manufacturer.

The ISO 11561 aging rates of the XPS samples were 1.5~2.2% different to those determined via EN 13164. The ISO 11561 standard assumes that the thermal resistance value measured at 91 days from the test start date is the thermal resistance value after 25 years [21]. In contrast, the EN 13164 standard specifies a correction of the thermal conductivity to account for damaged surfaces of the XPS samples without skin by subtracting 0.0007 W·m^−1^·K^−1^ from the measured aged thermal conductivity [18].

The decrease rate of thermal resistance in the PIR samples measured according to EN 13165 was 6% higher than that determined by Procedure B 175 days from the start. The change rate of thermal resistance according to EN 13165 was, on average, 3.4% higher than that determined by Procedure B 295 days from the start. In addition, the change rate of PIR_SY according to ISO 11561 was similar to that of EN 13165, while for PIR_JW, the ISO 11561 result was a little different to that determined according to EN 13165.

For PF_G, the result of Method 1 (slicing) was 19.3%, and those of Method 2 (heat aging) at 70 and 110 °C were 8.8% and 11.0%, respectively, according to EN 13166. For Method 1, the thermal conductivity of the assembly 46–52 days from the start date was used depending on the thickness of the PF_G sample. In the case of the PF_S sample, the thermal conductivity of the assembly 88–92 days from the start date was used because of the thickness of the specimen before slicing. Cutting the board sample into test specimens (slices) increases the open cell content on the surface of the test material, which reduces the effective thickness of the test specimens. To correct for the effective thickness, the initial thermal conductivity of the original specimen is subtracted from the initial thermal conductivity value of the assembled slices [20]. In the result of PF_G, the rate of decrease in thermal resistance was higher when the slicing acceleration of EN 13166 was applied than that determined by ISO 11561, and the opposite trend was analyzed for PF_S. Method 2 required a fixed increment depending on the blowing agent type and anti-diffusion facing, resulting in a thermal resistance aging value for PF_G that was 4.0% and 4.5% higher than those obtained for Procedures A and B.

In summary, even with the same PF material, the rate of change in thermal resistance could be different for each method, and it was determined that slicing acceleration promotes aging more than heat acceleration. Heat acceleration can take 21 to 175 days depending on the temperature conditions, while slicing acceleration requires 49 to 52 days for a product with a thickness of 70 mm, and 88 to 92 days for a product 60 mm thick.

Therefore, an appropriate method could be selected by considering the required time and cost, but phenol foam users should be aware of the variation in results due to heat and slicing. 

## 4. Discussion

The standards for each foam insulation material stipulate accelerated aging procedures via heat or slicing to determine their aging values. For all the samples, slicing acceleration resulted in a higher rate of degradation in thermal resistance than heat acceleration. However, when the same foam material was calculated according to the ISO 11561 and EN standards, the aged thermal performance change rates were similar overall.

**Figure 6:**
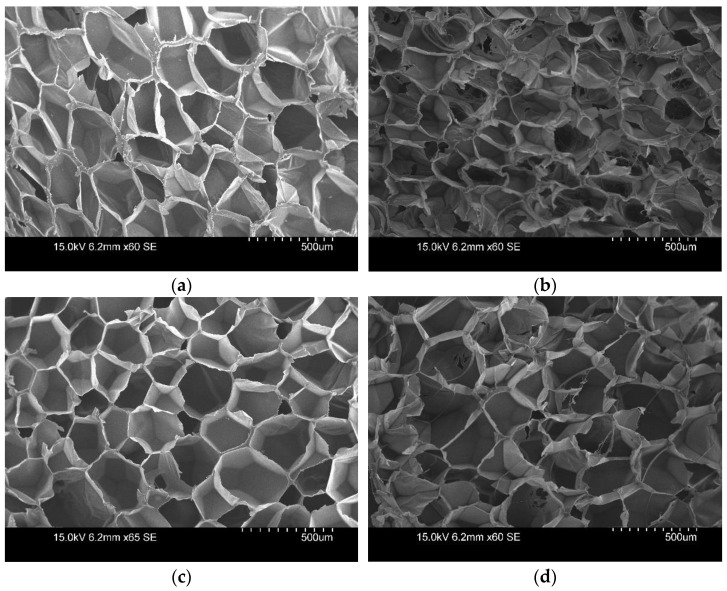
Scanning electron microscope (SEM) images of XPS samples: (**a**) before the heat acceleration of XPS_MI_S, (**b**) after the heat acceleration of XPS_MI_S, (**c**) before the heat acceleration of XPS_MI_1, and (**d**) after the heat acceleration of XPS_MI_1.

Figure 6 shows the effects of thermal acceleration on the XPS sample via scanning electron microscope (SEM) images. Figure 6a,c represent the initial cell structure of the XPS sample, and Figure 6b,d represent the cell structure after the heat acceleration. These images show a trend in foaming materials whereby the structure of the cell wall was maintained after heat acceleration, but most of walls appeared weak and lots of cells were open.

In any closed-cell product, there will be some finite, albeit small, fraction of open cell walls. However, when the sample is sliced at 10 mm, all the cells at the cut line are immediately open to the environment. This portion of the foam thickness is therefore immediately full of atmospheric gases and devoid of low-conductivity blowing agents [15,36]. It appears that slicing acceleration opens the cells more easily than heat acceleration, and this further promotes a decrease in the thermal resistance.

The blowing agent remains inside the cell and is replaced by air over time. In the acceleration procedure, the relative weight of XPS and PF decreased, because the HCFC, cyclopentane, and isopentane spread to the atmosphere due to the cells opening. A relative weight change in PIR also occurred when the cells were opened and the foaming agent (cyclopentane) diffused into the atmosphere [37]. There was little change in the PIR weight according to the heat acceleration method, but the relative weight of PIR samples according to the slicing acceleration method decreased by 8 to 9%. It might be that the effect of slicing acceleration is greater than the effect of thermal acceleration on the opening of the cell. In order to analyze the effect of additional thermal resistance degradation, future studies should secure experimental values for the diffusion coefficient of foaming gas and the amount of residual foamed gas.

**Figure 7:**
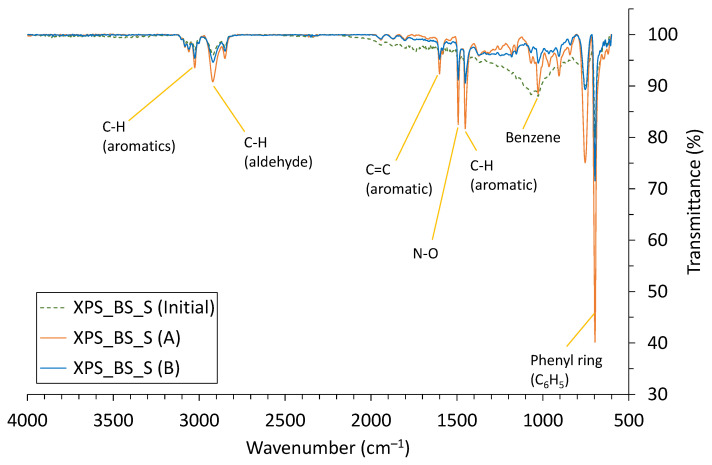
Fourier transform infrared spectroscopy (FTIR) of XPS_BS_S sample.

In addition, Fourier transform infrared spectroscopy (FTIR) was used to confirm the oxidation reaction of the foamed material due to heat acceleration. Figure 7 shows the transmission spectrum for the initial (non-aged) XPS sample and those aged at 110 °C and 70 °C. After the heat acceleration, XPS showed corresponding vibrational peaks because of thermal oxidation. Values of 680~1050 cm^−1^ correspond to the phenyl ring (C_6_H_5_) and benzene ring, and values of 1450~1600 cm^−1^ correspond to C-H, N-O, and C=C bonds. The phenyl ring (C_6_H_5_) was generated due to oxidation, and the intensity increased more when aged at 110° than when aged at 70°. Additionally, the intensity at peaks of 2850, 2920, and 3030 cm^−1^ increased, which indicates the presence of C-H aromatics and aldehyde. These are generated gradually due to thermal acceleration [38,39,40].

Heat acceleration could cause depolymerization, chain scission of the polymer matrix itself, chain shortening and softening, and a reaction in the amorphous structure to occur, thereby increasing the crystallinity, because a more crystalline polymer has more regularly aligned chains. Main-chain scission together with breakage and the re-forming of cross-links occurs during aging. At the same time, the thermal resistance might be decreased because the phonon scattering of the polymer is reduced [11,41,42,43].

## 5. Conclusions

In this study, we investigated the thermal properties of the foamed insulating materials XPS, PIR, and PF under the same test temperature conditions, and evaluated their aging values according to standardized aging procedures defined by EN and ISO standards. The purpose of this study was to clarify the change in thermal resistance for each material according to different accelerated aging procedures. The main conclusions drawn are as follows.

- According to the SEM images of the cell structure initially and after heat exposure, this method of acceleration made most of cell structures’ walls weak, and lots of cells were open. In addition, it could have accelerated the oxidation reaction of the foamed material. As a result, the cells in the foaming material subjected to heat acceleration might have more easily diffused the foaming agent into the atmosphere than those in the material at room temperature.
- In the acceleration procedure, the relative weight and density of the foam materials decreased because the HCFC, cyclopentane, and isopentane spread to the atmosphere due to the cells opening. Although a change in weight in response to the heat acceleration method occurred over time, the relative weight of the samples following the slicing acceleration method decreased very rapidly. It might be that the effect of slicing acceleration is greater than the effect of thermal acceleration on the opening of the cell.
- Upon comparing the aged values according to ISO and EN standards to the initial values, the analysis showed a change rate of 23 to 26% in PIR and 18 to 20% in PF. In XPS, a rate of change of 10 to 23.8% was calculated. There was a difference in the rate of change depending on the manufacturer and product group.
- Aging at 110 °C could be applied to PIR and PF, but not to XPS due to deformation. Slicing acceleration induced a thermal resistance reduction rate about three times faster than aging at 70 °C. However, the long-term changed thermal resistance values of the foam insulation material applied via the calculating procedure specified in the ISO and EN standards were similar.

## Figures and Tables

**Table 1:** Minimum performance standards for the building foam insulation materials XPS, PIR, and PF according to Korean standards.

Classification			Apparatus Density [d, kg∙m^−3^]	Initial Thermal Conductivity at Mean Temp. [λ, W∙m^−1^·K^−1^]	Bending Load at Break (Higher Than) [N]	Compressive Stress (Higher Than)	Water Absorption (Lower Than)
KS M 3808 (XPS)	Special		N/A	λ ≤ 0.027 at 23 ± 2 °C	45	25 [N∙cm^−2^]	146 [ng∙(m^2^·s·Pa)^−1^]
	No. 1		N/A	λ ≤ 0.028 at 23 ± 2 °C	35	15 [N∙cm^−2^]	
KS M 3809 (PIR)	1st class	No. 3	d ≥ 25	λ ≤ 0.025 at 20 ± 5 °C	15	10 [N∙cm^−2^]	3.0 [g∙100 cm^−2^]
	2nd class	No. 2	d ≥ 35	λ ≤ 0.023 at 20 ± 5 °C	25	10 [N∙cm^−2^]	
KS M ISO 4898 (PF)	1st class	A	d ≥ 30	λ ≤ 0.022 at 23 °C	15	6 [N∙cm^−2^]	4 [%(*v*/*v*)]

**Table 2:** Names of foam insulation material samples as determined by heat and slicing procedures.

Foam Insulation Material	Manufacturer	Thickness	Product Type Based on Korean Standards	Aging Procedure	Sample Name
Extruded polystyrene (XPS)	BS	50 mm	No. S, No. 1	A, B, C, D	XPS_BS_S or 1 (A~D)
	MI	50 mm	No. S, No. 1	A, B, C	XPS_MI_S or 1 (A~C)
Polyisocyanurate (PIR)	SY	90 mm	1st class—No. 3, 2nd class—No. 2	A, B, C, D	PIR_SY_13 or 22 (A~D)
	JW	90 mm	1st class—No. 3, 2nd class—No. 2	A, B, C	PIR_JW_13 or 22 (A~C)
Phenol foam (PF)	G	70 mm	1st class—No. A	A, B, C, D	PF_G (A~D)
	S	60 mm	1st class—No. A	A, B, C, D	PF_S (A~D)

**Table 3:** Initial and aging thermal resistance values [m^2^·K·W^−1^] according to different aging procedures.

Sample Name	Classification	No Standard (Procedure)				ISO 11561	EN 13164	EN 13165	EN 13166		
		A (110 °C)	B (70 °C)	C (Slicing)	D (Ambient Condition)	Slicing	Slicing	70 °C	110 °C	70 °C	Slicing
XPS_ BS_S	Initial	-	1.969	1.923	2.027	1.923	1.923	-	-		
	Aging	-	1.894	1.736	1.908	1.736	1.779	-	-		
	Rate	-	3.8%	9.7%	5.9%	9.7%	7.5%	-	-		
XPS_ BS_1	Initial	-	1.946	1.887	2.050	1.887	1.887	-	-		
	Aging	-	1.852	1.672	1.938	1.672	1.712	-	-		
	Rate	-	4.8%	11.4%	5.5%	11.4%	9.3%	-	-		
XPS_ MI_S	Initial	-	2.165	1.818	-	1.818	1.818	-	-		
	Aging	-	1.667	1.385	-	1.385	1.412	-	-		
	Rate	-	23.0%	23.8%	-	23.8%	22.3%	-	-		
XPS_ MI_1	Initial	-	2.058	1.639	-	1.639	1.639	-	-		
	Aging	-	1.613	1.374	-	1.374	1.401	-	-		
	Rate	-	21.6%	16.2%	-	16.2%	14.5%	-	-		
PIR_ SY_13	Initial	4.286	4.412	4.245	4.479	4.245	-	4.412	-		
	Aging	3.814	3.516	3.261	3.838	3.261	-	3.346	-		
	Rate	11.0%	20.3%	23.2%	14.3%	23.2%	-	24.2%	-		
PIR_ SY_22	Initial	4.327	4.412	4.186	4.380	4.186	-	4.412	-		
	Aging	3.689	3.516	3.180	3.750	3.180	-	3.333	-		
	Rate	14.7%	20.3%	24.0%	14.4%	24.0%	-	24.5%	-		
PIR_ JW_13	Initial	4.167	4.390	4.500	-	4.500	-	4.390	-		
	Aging	3.982	3.488	3.321	-	3.321	-	3.346	-		
	Rate	4.4%	20.5%	26.2%	-	26.2%	-	23.8%	-		
PIR_ JW_22	Initial	4.478	4.412	4.500	-	4.500	-	4.412	-		
	Aging	4.167	3.543	3.358	-	3.358	-	3.396	-		
	Rate	6.9%	19.7%	25.4%	-	25.4%	-	23.0%	-		
PF_G	Initial	3.571	3.763	3.241	3.737	3.241	-	-	3.571	3.763	3.483
	Aging	3.398	3.349	2.652	3.544	2.652	-	-	3.256	3.349	2.811
	Rate	4.8%	11.0%	18.2%	5.2%	18.2%	-	-	8.8%	11.0%	19.3%
PF_S	Initial	-	-	2.871	3.170	2.871	-	-	-	-	2.871
	Aging	-	-	2.299	2.900	2.299	-	-	-	-	2.299
	Rate	-	-	19.9%	8.5%	19.9%	-	-	-	-	19.9%

## Abbreviations

EPS	expanded polystyrene
XPS	extruded polystyrene
PF	phenol foam
PIR	polyisocyanurate
PS	polystyrene
PU	polyurethane
